# Supplementary material for: Efficacy of Environmental Cleaning Protocol Featuring Real-Time Feedback with and without PX-UV in Reducing the Contamination of Gram-Negative Microorganisms on High-Touch Surfaces in Four Intensive Care Units in Thailand
Source: Antibiotics (Basel). 2023 Feb 22;12(3):438. doi: 10.3390/antibiotics12030438 (PMC10044567; doi:10.3390/antibiotics12030438)
Supplement: Supplementary file 1 [file antibiotics-12-00438-s001.zip › antibiotics-2204602-supplementary.pdf]

Supplementary 1: Visual List and Description of High-Touch Surfaces in the Intensive Care Unit

**Sampling areas:**<br>• Designated area to be sampled Before PX-UV (Red)<br>• Designated area to be sampled After PX-UV (Blue)	
**Infusion pump**<br>• Area on top of the infusion pump bottom.<br>• Before PX-UV: Sample the left area on top of the infusion pump bottom.<br>• After PX-UV: Sample the right area on top of the infusion pump bottom.<br>•	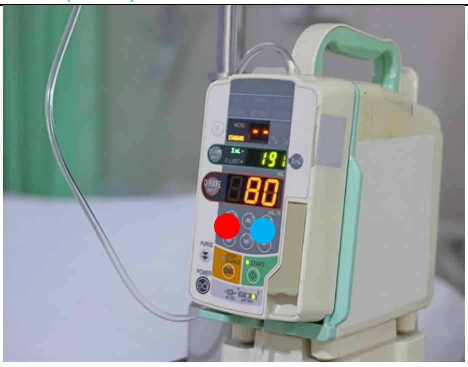
**Overbed Table**<br>• Area on top (facing the ceiling) of the middle of the table<br>• Before PX-UV: Sample the left top area of the overbed table<br>• After PX-UV: Sample the right top area of the overbed table	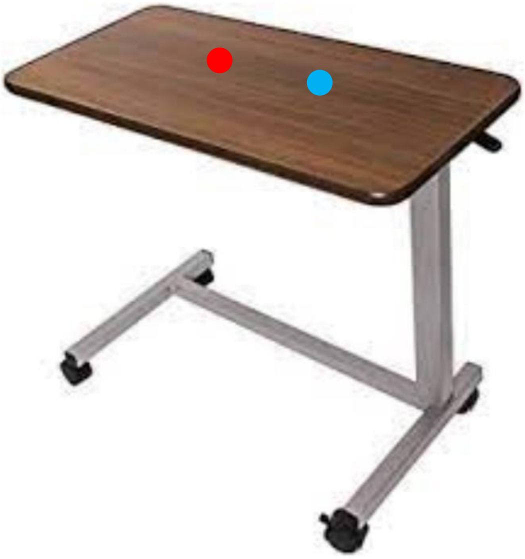
**Bedside Table**<br>• Area on top (facing the ceiling) of the middle of the table<br>• Before PX-UV: Sample the left top area of the bedside table<br>• After PX-UV: Sample the right top area of the bedside table	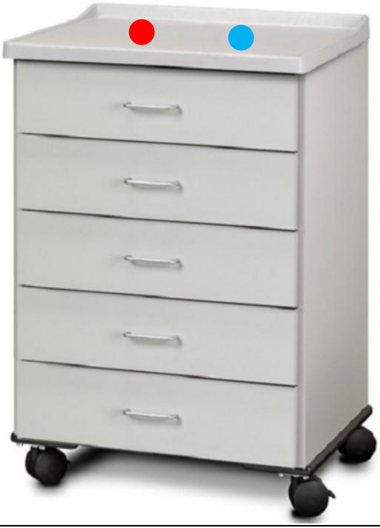

**Medication Cart**<br>• Midpoint area facing the sampler of the medication cart<br>• Before PX-UV: Sample the left-side area of the bedside stand below the drawer handle<br>• After PX-UV: Sample the right-side area of the bedside stand below the drawer handle	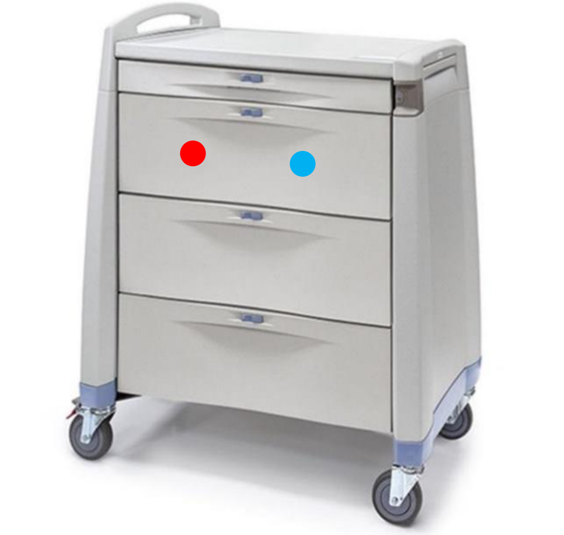
**Vital Signs Screen**<br>• Area facing the sampler in the bottommost region of the controls along the screen edge<br>• Before PX-UV: Sample the area adjacent to mid-line of the screen edge<br>• After PX-UV: Sample the right-side area of the midpoint before buttons region along the screen edge	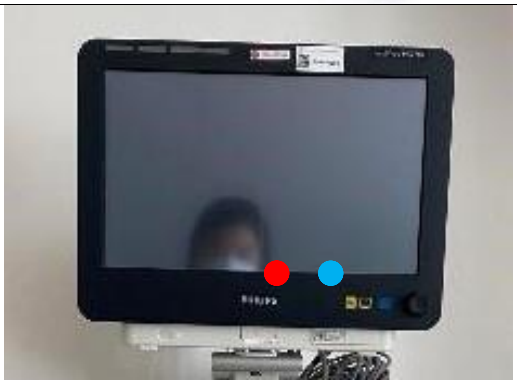
